# Wm. P. Morgan, D.D.S.

**Published:** 1915-01

**Authors:** 


### OBITUARY.

WM. P. MORGAN, D.D.S.—Dr. Wm. P. Morgan, of Saginaw, Michigan, died at his home in Saginaw December 21, 1914, from an attack of pneumonia. He was 68 years old at the time of his death. He was one of the oldest and best known and loved dentists of Michigan.

Dr. Morgan was born October 8, 1846, in Albion, N. Y. He graduated from Harvard Dental School in 1869 and began practice in Saginaw in the latter part of 1870, where he practiced continuously until May, 1913, when he gave up practice because of impaired health. Dr. Morgan always took a keen interest in the profession, he was a constant attendant on all meetings of the State Society, and held practically all the offices. He was a quiet and unobtrusive gentleman, with strong moral and religious convictions. He was an influential member of the Baptist Church and greatly interested in the work of the Young Men's Christian Association, and an active worker in the cause of temperance. His father was a farmer in New York State and he has all his life been much interested in farming, which was his principle diversion. He gave much time to the development of his farm and of the interests of farmers in his section of the State.

Although a quiet and peaceful man he accomplished much in his life. He was not only a perfect gentleman, but an enthusiastic professional man, and he will be greatly missed in his home city and throughout the State. He never was a man of rugged health, and this probably explains the fact that dental societies have not seen much of him the last few years, because he has not been physically able to get out to the meetings.

Dr. Morgan had the unquestioned confidence of the people in his own city and will be generally missed. He leaves a widow, two sons and two daughters to mourn his loss, which to them is great indeed.

